# Multilevel Determinants of Acromiohumeral Distance Within a Systems-Based Functional Morphology Framework and Their Implications for Personalized Neuromusculoskeletal Modelling

**DOI:** 10.3390/jfmk11030314

**Published:** 2026-08-12

**Authors:** Luis Alfonso Arráez-Aybar, Carlos Miquel García-de-Pereda-Notario, Luis Palomeque-Del-Cerro, Juan José Montoya-Miñano

**Affiliations:** 1 Department of Anatomy and Embryology, School of Medicine, Complutense University of Madrid, 28040 Madrid, Spain; arraezla@med.ucm.es; 2 UCM Research Group No. 920202, School of Medicine, Complutense University of Madrid, 28040 Madrid, Spain; 3 Department of Physiotherapy, School of Nursing and Physiotherapy “Salus Infirmorum”, Pontifical University of Salamanca, 28015 Madrid, Spain; lpalomequede@upsa.es; 4 Escuela de Osteopatía de Madrid, 28033 Madrid, Spain; 5 Department of Radiology and Rehabilitation, School of Medicine, Complutense University of Madrid, 28040 Madrid, Spain; jjmontoy@ucm.es

**Keywords:** acromiohumeral distance, functional morphology, shoulder biomechanics, personalized neuromusculoskeletal modelling, systems theory, emergence, movement variability, neuromuscular control, connective tissue, digital twin

## Abstract

**Background:** The acromiohumeral distance (AHD) is a widely used imaging parameter for evaluating the subacromial region and shoulder function. Traditionally, it has been interpreted as a static anatomical measurement reflecting the available subacromial space and associated with rotator cuff pathology, superior humeral migration, and subacromial impingement. However, inconsistent relationships between AHD, symptoms, and function suggest that purely structural interpretations may be insufficient. **Objectives:** To propose a systems-based functional morphology framework that reinterprets AHD as an emergent functional state variable reflecting the instantaneous state of the glenohumeral system. **Methods:** A conceptual synthesis integrated evidence from shoulder biomechanics, functional morphology, movement science, connective tissue research, systems theory, and personalized neuromusculoskeletal modelling. Structural, connective tissue, neuromuscular, and mechanical determinants of AHD were integrated within a multilevel systems perspective. **Results:** The proposed framework conceptualizes AHD as emerging from the dynamic interaction of four organizational domains: structural constraints, connective tissue properties, neuromuscular regulation, and mechanical context. Rather than representing a fixed anatomical space, AHD is interpreted as a potential systems-level functional biomarker whose value varies according to the functional state of the glenohumeral system. This perspective explains the variability observed across imaging studies and the frequent discordance between structural findings, symptoms, and functional outcomes. **Conclusions:** Reframing AHD as an emergent functional state variable provides a theoretical basis for integrating anatomy, biomechanics, movement behavior, and neuromuscular control within personalized shoulder assessment. This systems-based perspective may help bridge the traditional gap between structural imaging and clinical presentation while supporting future developments in precision rehabilitation and subject-specific computational modelling.

## 1. Introduction

The acromiohumeral distance (AHD), defined as the distance between the inferior aspect of the acromion and the superior aspect of the humeral head, is one of the most frequently used imaging parameters for evaluating the subacromial region and shoulder function [1,2]. Traditionally, AHD has been interpreted as a structural indicator of the available subacromial space, with reductions in this distance commonly associated with superior humeral migration, rotator cuff pathology, and subacromial impingement mechanisms [3,4,5,6].

This structural interpretation has significantly influenced both clinical assessment and therapeutic decision-making. Acromial morphology, coracoacromial arch configuration, and superior displacement of the humeral head have frequently been regarded as primary determinants of subacromial clearance and tissue loading [5,7,8]. However, despite the widespread use of AHD as a diagnostic and prognostic parameter, growing evidence suggests that its relationship with pain, symptoms, and functional performance is considerably more complex than originally proposed [9].

Several observations challenge purely structural interpretations of AHD. First, substantial overlap exists between imaging findings observed in symptomatic and asymptomatic individuals, and structural abnormalities frequently demonstrate weak associations with pain and disability [9,10]. Second, AHD demonstrates significant variability according to arm position, loading conditions, movement strategy, and neuromuscular activation patterns [2,11]. Third, dynamic studies indicate that subacromial organization changes continuously during movement, suggesting that static measurements may capture only a limited representation of a highly adaptive biomechanical system [1,12].

Collectively, these findings do not diminish the clinical relevance of AHD but indicate that its interpretation requires a broader conceptual perspective. Although structural anatomy undoubtedly establishes the physical boundaries within which shoulder function occurs, structural variables alone appear insufficient to explain the marked variability observed across individuals and functional conditions. Consequently, an important conceptual gap remains: despite extensive research on the anatomical and biomechanical determinants of AHD, no integrative framework has yet explained how structural, connective tissue, neuromuscular, and mechanical factors interact to determine its instantaneous functional expression.

These findings are consistent with contemporary perspectives in functional morphology and systems science, which increasingly recognize that biological behavior emerges from interactions among multiple organizational levels rather than from isolated anatomical structures [13,14]. Within this framework, musculoskeletal function is understood as the product of dynamic relationships between morphology, connective tissue architecture, neuromuscular regulation, and mechanical context. From this perspective, clinically relevant variables may be better interpreted as emergent properties of interacting biological systems than as direct expressions of individual anatomical components.

Recent developments in movement science further support this interpretation. Dynamical systems theory emphasizes that movement variability is not merely random noise but an adaptive property that contributes to robustness and functional flexibility [15,16]. More broadly, dynamical systems approaches describe biological movement as a self-organizing process in which stable behavioral patterns emerge from interactions among multiple system components and changing task constraints [17,18]. Within this perspective, functional variables may be interpreted as expressions of evolving system states rather than as fixed properties of individual anatomical structures. Similarly, the theory of motor synergies proposes that stable movement outcomes emerge from coordinated interactions among multiple biomechanical and neuromuscular components rather than from the isolated control of individual structures [19]. These concepts suggest that the position of the humeral head and the organization of the subacromial region are likely influenced by distributed regulatory processes extending beyond local anatomical morphology.

Contemporary anatomical research also highlights the importance of connective tissue continuity and myofascial force transmission in musculoskeletal organization.

Rather than functioning as independent mechanical units, muscles, tendons, fasciae, ligaments, and capsules form interconnected networks capable of transmitting and redistributing forces throughout the system [20,21]. Emerging evidence from fascial anatomy and connective tissue research increasingly supports this integrated view of musculoskeletal organization, although the specific contribution of these mechanisms to subacromial biomechanics remains incompletely understood. Complementary biotensegrity models further propose that stability emerges from the balance between continuous tensile forces and discontinuous compressive elements distributed across the musculoskeletal system [22,23]. Although biotensegrity should currently be regarded as a theoretical model rather than a fully validated biomechanical paradigm, it offers a valuable systems-based perspective for understanding how distributed mechanical interactions may contribute to shoulder organization beyond purely local structural relationships. These perspectives suggest that subacromial organization may depend on global force-distribution mechanisms rather than solely on local structural characteristics.

**Figure 1:**
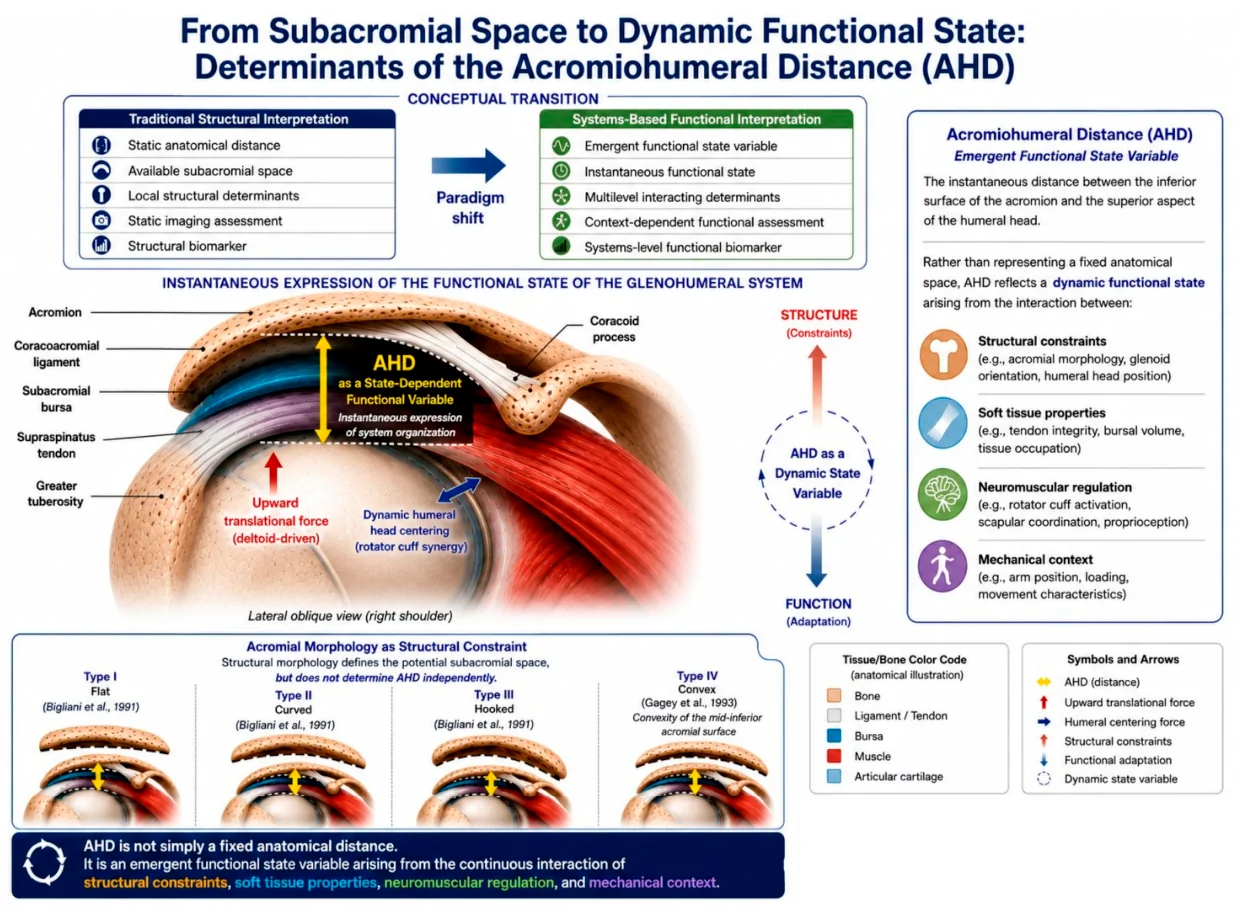
**Conceptual transition from the traditional structural interpretation of the acromiohumeral distance (AHD) to a systems-based functional morphology framework.** The upper panel summarizes the paradigm shift from a static anatomical interpretation of AHD as a measure of subacromial space to its conceptualization as an emergent functional state variable reflecting the instantaneous functional state of the glenohumeral system. The central illustration depicts the principal structural, connective tissue, neuromuscular, and mechanical determinants that continuously interact to shape AHD. The lower panel highlights the role of acromial morphology as an important structural constraint that influences, but does not independently determine, the functional expression of AHD [8,24]. Arrows indicate directions of forces and functional influences: yellow = AHD (distance), red = upward translational force (deltoid-driven), blue = dynamic humeral head centering (rotator cuff synergy); vertical gradient arrows represent structural constraints (upward) and functional adaptation (downward). Colors represent main anatomical tissues as indicated in the color code.

Applied to shoulder biomechanics, these developments support a broader interpretation of AHD as a dynamic variable reflecting the instantaneous functional state of the glenohumeral system. As illustrated in Figure 1, the subacromial region is influenced by the interaction of structural morphology, connective tissue properties, neuromuscular stabilization, and mechanical loading conditions. Under this perspective, AHD should not be viewed as a fixed anatomical distance, but rather as a state-dependent expression of system organization. Consequently, the functional significance of a given AHD value depends not only on its absolute magnitude but also on the organizational context from which it emerges. This systems-based interpretation provides a coherent explanation for the variability observed across imaging studies and for the frequently reported dissociation between structural findings and clinical presentation.

The purpose of the present work is to propose a systems-based functional morphology framework for understanding the acromiohumeral distance as an emergent functional state variable. Rather than presenting new experimental data, this Perspective develops an integrative conceptual synthesis that brings together current evidence from functional anatomy, shoulder biomechanics, movement science, connective tissue research, systems theory, and personalized neuromusculoskeletal modelling into a unified explanatory model. Building on this body of evidence, we propose a conceptual model in which AHD reflects multilevel interactions across anatomical, biomechanical, neuromuscular, and contextual domains. Specifically, AHD is interpreted as an emergent functional state variable arising from the continuous interaction among four organizational domains: structural constraints, connective tissue properties, neuromuscular regulation, and mechanical context. This systems-based approach provides a theoretical basis for interpreting AHD beyond reductionist structural paradigms while supporting future developments in personalized assessment, rehabilitation, and computational modelling of shoulder function.

## 2. Conceptual Synthesis and Theoretical Framework

### 2.1. Conceptual Synthesis Methodology

This Perspective presents an integrative conceptual framework rather than reporting new experimental data or performing a systematic review. The proposed framework was constructed through a conceptual synthesis of current concepts and evidence drawn from complementary disciplines, following established principles for conceptual framework development in multidisciplinary research [25]. The aim was to integrate existing knowledge into a coherent explanatory model capable of providing a broader interpretation of the AHD within a systems-based functional morphology perspective.

The proposed framework was developed from the recognition that AHD has traditionally been interpreted within a predominantly structural paradigm, whereas accumulating evidence from anatomy, biomechanics, movement science, and clinical research indicates that its behaviour cannot be fully explained by isolated anatomical variables alone. Accordingly, the objective was not to provide an exhaustive review of the literature but to integrate well-established concepts from complementary disciplines into a coherent systems-based interpretation capable of explaining the dynamic and context-dependent behaviour of AHD.

To achieve this objective, current concepts and evidence from six complementary knowledge domains were critically integrated: (i) functional anatomy and shoulder morphology, (ii) shoulder biomechanics, (iii) connective tissue and myofascial organization, (iv) neuromuscular control and movement science, (v) dynamical systems and complex systems theory, and (vi) personalized neuromusculoskeletal modelling. These domains were selected because each contributes an established theoretical and/or experimental perspective on factors known to influence shoulder function and, collectively, they provide a broader understanding than any discipline considered independently.

Rather than organizing the available knowledge according to traditional disciplinary boundaries, the evidence was integrated into four interacting organizational domains representing complementary analytical perspectives within a single biological system: structural constraints, connective tissue properties, neuromuscular regulation, and mechanical context. This classification is intended as a conceptual framework that facilitates the interpretation of multilevel interactions rather than as a representation of independent biological compartments. In vivo, these domains continuously interact and mutually influence one another. Accordingly, their separate presentation in the following sections is intended solely for explanatory purposes and does not imply functional independence.

Within this framework, the concept of emergence is adopted from complex systems theory. An emergent property is understood as a system-level characteristic that arises from the dynamic interactions among multiple interconnected components and cannot be fully explained by examining any individual component in isolation [13,14]. Rather than representing the additive contribution of isolated variables, emergent properties reflect patterns of organization that develop across interacting levels of a biological system. Applied to shoulder biomechanics, this perspective suggests that AHD should be interpreted as the instantaneous expression of the functional state of the glenohumeral system rather than as the direct consequence of any single anatomical determinant.

Consequently, the following sections examine each organizational domain separately for analytical clarity while recognizing that shoulder function emerges from their continuous interaction. The proposed framework therefore represents a conceptual model intended to integrate current knowledge, generate testable hypotheses, and provide a theoretical foundation for future experimental research and the development of personalized neuromusculoskeletal models. Based on this conceptual synthesis, the following sections describe how interactions among these four organizational domains give rise to AHD as an emergent functional state variable.

### 2.2. The Acromiohumeral Distance as an Emergent Functional State Variable

Building upon the conceptual synthesis described in the previous section, the AHD is proposed as an emergent functional state variable rather than as a purely structural anatomical measurement. Within the proposed systems-based functional morphology framework, AHD is understood as the instantaneous expression of the functional organization of the glenohumeral system, arising from the continuous interaction among anatomical, connective tissue, neuromuscular, and mechanical determinants rather than from any single structural component [1,2,13,14,15,16,17,18,19].

This interpretation does not challenge the established importance of anatomical morphology in shoulder biomechanics. Structural characteristics—including acromial morphology, coracoacromial arch configuration, glenoid orientation, humeral head position, and rotator cuff integrity—define the anatomical boundaries within which shoulder function occurs and contribute substantially to the mechanical organization of the subacromial region [3,4,5,6,7,8,24]. However, increasing evidence indicates that structural variables alone do not adequately explain the substantial variability observed in AHD measurements, nor their frequently inconsistent relationship with pain, symptoms, and functional performance [9,10,11,12].

Accordingly, AHD should not be interpreted as the direct consequence of any single determinant but rather as the **system-level outcome** of multiple interacting processes operating across different organizational levels. Within this framework, structural anatomy provides the physical constraints of the system, whereas the observed AHD at any given moment reflects the integrated influence of connective tissue behaviour, neuromuscular regulation, and the prevailing mechanical context [13,14,15,16,17,18,19,20,21,22,23]. Consequently, similar anatomical configurations may generate different AHD values under different functional conditions, whereas comparable AHD measurements may emerge from distinct underlying biomechanical organizations [9,10,11,12,15,16,17,18,19].

For conceptual clarity, these determinants are organized into four interacting organizational domains: (i) structural constraints, (ii) connective tissue properties, (iii) neuromuscular regulation, and (iv) mechanical context. These domains do not represent independent biological compartments but complementary analytical perspectives that facilitate the interpretation of multilevel interactions within a single integrated biological system. Their distinction is therefore methodological rather than biological, allowing each domain to be examined individually while recognizing that, in vivo, they continuously interact and mutually influence one another.

This systems-based interpretation also provides a coherent explanation for several observations that remain difficult to reconcile within exclusively structural models. These include the marked variability of AHD across different arm positions and functional tasks, the influence of loading conditions and movement strategies, the overlap of imaging findings between symptomatic and asymptomatic individuals, and the frequently reported dissociation between static imaging measurements and clinical presentation [1,2,9,10,11,12]. Rather than viewing these findings as contradictory or simply reflecting measurement variability, the proposed framework interprets them as expected consequences of the adaptive behaviour of a complex biological system [13,14,15,16,17,18,19].

**Figure 2:**
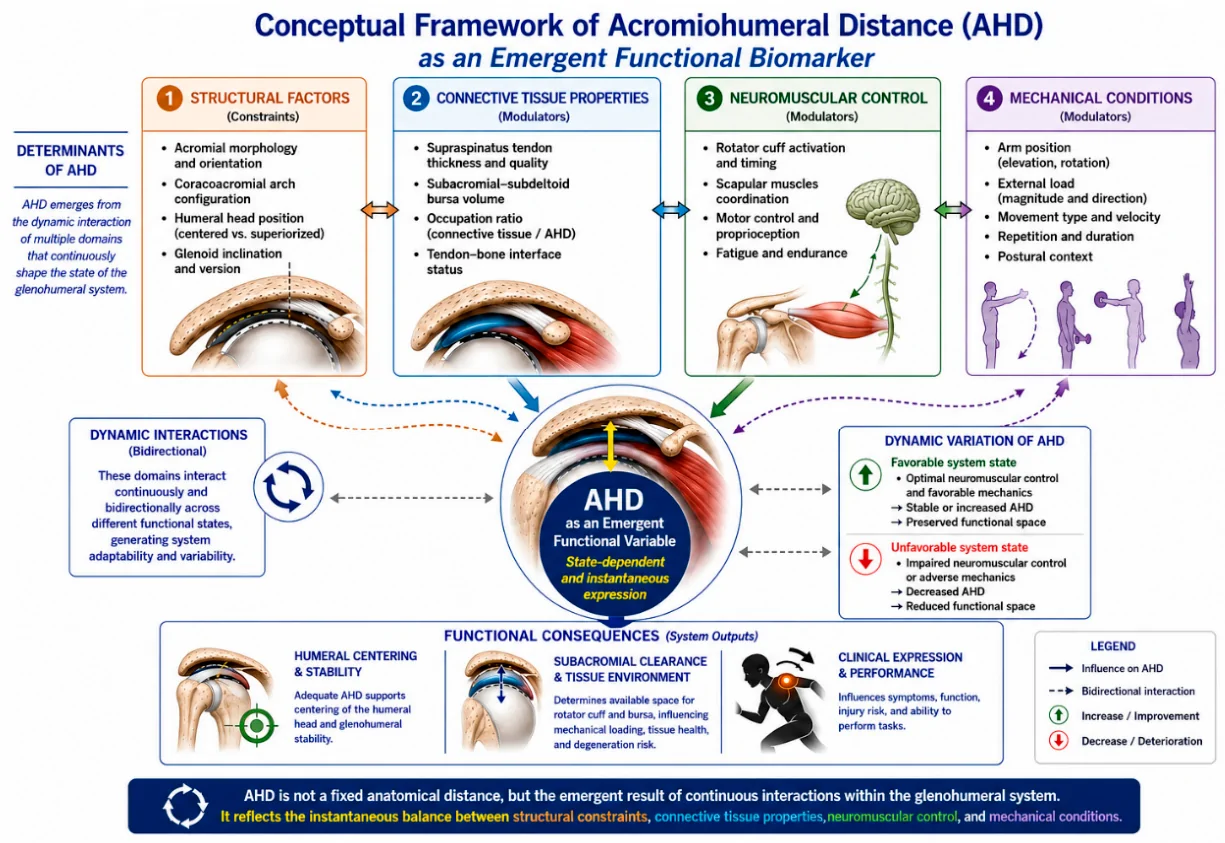
**Conceptual framework of the acromiohumeral distance (AHD) as an emergent functional biomarker of the glenohumeral system.** Systems-based representation of the multilevel interactions among structural morphology, connective tissue properties, neuromuscular regulation, and mechanical conditions. Their continuous interaction gives rise to AHD as an emergent systems-level functional biomarker reflecting the instantaneous functional state of the shoulder complex.

The following sections examine each organizational domain separately for analytical purposes before integrating them into a unified conceptual framework. Figure 2 summarizes these multilevel interactions and illustrates how their continuous integration gives rise to AHD as an emergent functional state variable, providing the theoretical foundation for the systems-based interpretation and personalized neuromusculoskeletal modelling approach proposed in this Perspective.

### 2.3. Structural Constraints and Morphological Boundaries

Structural anatomy provides the physical framework within which shoulder function occurs. Acromial morphology, coracoacromial arch configuration, glenoid orientation, humeral head position, and the integrity of the rotator cuff establish relatively stable anatomical boundaries that influence the potential dimensions and mechanical organization of the subacromial region [3,4,5,6,7,8,24].

Within the proposed systems-based functional morphology framework, these anatomical characteristics are best interpreted as structural constraints that define the biomechanical landscape within which functional behaviour emerges, rather than as isolated determinants of shoulder function. Structural morphology establishes the range of mechanically feasible system configurations while allowing substantial adaptability through continuous interactions with connective tissue properties, neuromuscular regulation, and the prevailing mechanical context [13,14,15,16,17,18,19]. Consequently, anatomical structure provides the necessary physical substrate for shoulder function without independently determining its instantaneous functional state.

This interpretation is supported by clinical observations demonstrating that similar structural configurations may be associated with markedly different functional performance, movement strategies, symptom profiles, and acromiohumeral distance measurements [9,10,11,12]. Likewise, individuals presenting comparable AHD values may differ substantially in neuromuscular organization, tissue behaviour, or mechanical loading conditions. These findings suggest that anatomical morphology should be regarded as an essential—but not sufficient—determinant of shoulder organization.

Accordingly, structural morphology should be interpreted as establishing the anatomical landscape within which adaptive and maladaptive functional states may emerge through interactions with the remaining organizational domains proposed in the present framework. This perspective preserves the central importance of anatomical structure while integrating it into a broader systems-based interpretation of shoulder biomechanics that better accommodates the dynamic and context-dependent behaviour of the acromiohumeral distance.

### 2.4. Connective Tissue Continuity and Myofascial Integration

The connective tissue system plays an important integrative role in the structural and functional organization of the musculoskeletal system. Contemporary anatomical research increasingly recognizes fasciae, tendons, ligaments, joint capsules, and surrounding connective tissues as components of a continuous mechanical network capable of transmitting, redistributing, and modulating mechanical forces across multiple anatomical regions [20,21,26,27]. Rather than functioning as isolated structures, these tissues contribute to the structural and mechanical continuity that characterizes normal musculoskeletal behaviour.

Within the shoulder complex, rotator cuff tendons, the subacromial–subdeltoid bursa, joint capsule, and surrounding connective tissues contribute to the organization of the subacromial environment. Beyond occupying physical space, these structures influence tissue deformation, load transmission, mechanical coupling, and the adaptive response of the glenohumeral system during movement. The relative contribution of each of these components is likely to vary according to the functional demands imposed on the shoulder and the mechanical context in which movement occurs. Moreover, these interactions are unlikely to remain constant across individuals, further supporting the need for individualized interpretations of shoulder function.

Biotensegrity concepts provide a complementary systems-based perspective by proposing that mechanical stability emerges from interactions between continuous tensile elements and discontinuous compressive structures distributed throughout the musculoskeletal system [22,23]. Although biotensegrity should currently be regarded as a theoretical biomechanical model whose empirical validation is still evolving, it offers a useful conceptual framework for understanding how distributed force transmission and mechanical interdependence may influence shoulder organization beyond purely local structural relationships. This interpretation should therefore be understood as complementary to, rather than replacing, established anatomical and biomechanical models.

Consequently, AHD may reflect not only osseous relationships but also the behaviour of interconnected connective tissue structures that contribute to the overall mechanical organization of the glenohumeral system. However, the precise contribution of connective tissue continuity and distributed force transmission to the dynamic regulation of AHD remains to be established through future experimental and computational studies [27]. Within the proposed framework, connective tissue continuity is therefore interpreted as one of the interacting organizational domains that contributes to the emergence of AHD as a functional state variable, rather than as an isolated explanatory mechanism.

### 2.5. Neuromuscular Coordination and Movement Variability

Neuromuscular regulation represents one of the principal mechanisms through which shoulder organization is continuously adapted to changing functional demands. Rotator cuff activation, scapular muscle coordination, proprioceptive feedback, motor planning, and fatigue management collectively contribute to the maintenance of glenohumeral stability during movement. These regulatory processes are inherently adaptive, allowing the shoulder to accommodate changing task requirements while maintaining functional stability across a wide range of mechanical conditions.

The theory of motor synergies proposes that the nervous system stabilizes functional outcomes through coordinated interactions among multiple biomechanical elements rather than through rigid control of individual structures [19]. Within this framework, humeral head centering emerges from the coordinated activity of numerous muscular and neural components acting as an integrated system. Accordingly, neuromuscular regulation contributes to the continuous stabilization of functional states rather than to the rigid control of individual anatomical structures.

Recent neurophysiological evidence further suggests that cortical representations of shoulder muscles are organized predominantly according to task demands rather than strictly according to individual muscles, supporting the view that shoulder function emerges from distributed neural control strategies rather than isolated muscular actions [28]. These findings reinforce the concept that shoulder organization depends on coordinated neural networks capable of adapting motor output to changing functional and mechanical demands.

Similarly, dynamical systems approaches emphasize that movement variability constitutes an adaptive characteristic of healthy biological systems rather than a source of error or instability [15,16,17,18]. Rather than representing biological noise, movement variability enables biological systems to maintain performance under changing environmental and mechanical conditions while preserving flexibility, robustness, and adaptability. From this perspective, variability is understood as an inherent property of self-organizing biological systems, reflecting their capacity to continuously adapt to internal and external constraints.

Applied to shoulder function, these perspectives suggest that fluctuations in AHD may be interpreted as expressions of adaptive neuromuscular regulation rather than merely as indicators of mechanical instability [15,16,17,18,19,28]. Within the proposed framework, the functional state of the subacromial region emerges from the dynamic interplay between anatomical structure, neuromuscular organization, connective tissue behaviour, and mechanical context, rather than from any single organizational domain acting in isolation. Consequently, variations in AHD should be interpreted within the broader context of integrated shoulder function, where neuromuscular coordination represents one of the principal mechanisms contributing to the emergence of AHD as a functional state variable.

### 2.6. Mechanical Context and Dynamic State Dependency

Shoulder biomechanics are strongly influenced by the mechanical conditions under which movement occurs. Arm position, external loading, movement velocity, task requirements, repetition, fatigue, and postural context all modify the forces acting on the glenohumeral system and therefore influence subacromial organization [2,11,29]. Rather than representing external influences acting independently of the biological system, these mechanical conditions continuously interact with anatomical structure, connective tissue behaviour, and neuromuscular regulation throughout movement.

These factors are inherently dynamic and vary continuously during functional activities. Consequently, the mechanical environment of the shoulder cannot be adequately characterized by static measurements obtained under a single testing condition. Instead, shoulder behaviour should be understood as state dependent, with distinct functional states emerging under different combinations of loading conditions, movement demands, and environmental constraints. The same anatomical configuration may therefore give rise to different functional expressions depending on the mechanical context in which it operates.

Within the proposed framework, mechanical conditions function as dynamic modulators that continuously influence the interaction among the structural, connective tissue, and neuromuscular organizational domains, thereby shaping the instantaneous expression of AHD. This interpretation is consistent with observations demonstrating substantial variation in subacromial dimensions during active movement and across different functional tasks [1,12]. Accordingly, AHD should not be interpreted as a fixed anatomical attribute but as a context-dependent expression of glenohumeral system organization.

From a systems perspective, mechanical context does not simply modify shoulder behaviour; it participates in the continuous reorganization of the system by altering the dynamic interplay among its organizational domains. Consequently, changes in AHD across different tasks or loading conditions may reflect adaptive transitions between functional states rather than isolated mechanical effects. This interpretation reinforces the concept of AHD as an emergent functional state variable whose significance depends on the integration of mechanical context with the remaining determinants of shoulder function.

### 2.7. Integration into Personalized Neuromusculoskeletal Models

The multidimensional organization of shoulder function suggests that future neuromusculoskeletal models should move beyond predominantly structural representations toward more integrative frameworks capable of incorporating the dynamic interactions among anatomical morphology, connective tissue behaviour, neuromuscular regulation, and mechanical context. Rather than representing these components as independent determinants, personalized models should account for their continuous and context-dependent interactions, recognizing that functional behaviour emerges from the organization of the system as a whole [30,31,32].

Traditional biomechanical models have substantially advanced the understanding of shoulder mechanics by characterizing anatomical geometry, muscle forces, and joint loading [33,34]. However, these approaches frequently simplify the dynamic complexity of biological systems by treating many relevant variables as fixed or independent. A systems-based perspective does not replace these established models but extends their interpretative capacity by incorporating adaptive interactions that occur across multiple organizational levels [30,31].

Recent developments in computational biomechanics, subject-specific modelling, and machine learning further support this transition toward personalized neuromusculoskeletal modelling. Machine learning approaches are increasingly being used to integrate imaging, motion analysis, electromyography, musculoskeletal simulations, and wearable sensor data, facilitating subject-specific predictions while reducing the computational complexity traditionally associated with personalized biomechanical modelling [35]. These developments provide a promising foundation for future digital-twin approaches capable of representing the dynamic behaviour of individual musculoskeletal systems.

Within this conceptual framework, AHD may serve as a potential systems-level functional biomarker reflecting the integrated behaviour of the glenohumeral system rather than the isolated contribution of any single anatomical or biomechanical factor. Its interpretation should therefore be considered within the broader context of the dynamic interplay among structural constraints, connective tissue continuity, neuromuscular coordination, and mechanical context, all of which may vary across individuals and functional tasks.

The progressive integration of advanced medical imaging, wearable technologies, motion analysis, computational biomechanics, and artificial intelligence offers new opportunities to develop personalized neuromusculoskeletal models capable of capturing these multidimensional interactions [30,31,32,35]. Although substantial methodological and computational challenges remain, integrating multilevel anatomical, biomechanical, and neurophysiological information provides a promising framework for improving both the interpretation of shoulder function and the development of personalized diagnostic, prognostic, and therapeutic strategies. Rather than predicting isolated structural outcomes, future neuromusculoskeletal models may ultimately characterize how functional states emerge, adapt, and reorganize across changing biological and mechanical conditions.

From a practical perspective, implementing AHD as a systems-level functional biomarker would require integrating structural imaging (e.g., radiography, ultrasound, or MRI), dynamic movement analysis, neuromuscular assessment (including electromyography where appropriate), and subject-specific biomechanical modelling [36,37]. Rather than relying on a single static measurement, AHD would be interpreted within the broader context of these complementary sources of information to characterize the functional state of the glenohumeral system.

### 2.8. Conceptual Synthesis

Collectively, the four organizational domains described above support a reinterpretation of the AHD as an emergent functional state variable rather than a purely structural anatomical measurement. Structural constraints define the biomechanical landscape within which shoulder function occurs, connective tissue continuity contributes to structural and mechanical integration, neuromuscular regulation continuously coordinates system behaviour, and mechanical context dynamically modulates the expression of these interactions during functional activity.

Importantly, these domains should not be interpreted as independent determinants acting in parallel. Rather, they constitute interdependent organizational levels whose continuous interactions give rise to the instantaneous functional state of the glenohumeral system. Consequently, the observed value of AHD should be understood as the integrated expression of system organization at a given moment, rather than as the direct consequence of any isolated anatomical, biomechanical, or neuromuscular variable.

This systems-based interpretation provides a coherent conceptual framework for explaining several observations that remain difficult to reconcile within reductionist structural models, including the variability of AHD across different functional tasks, the overlap between symptomatic and asymptomatic individuals, and the frequently reported dissociation between imaging findings and clinical presentation [1,2,9,10,11,12,13,14,15,16,17,18,19]. By emphasizing interactions rather than isolated determinants, the proposed framework offers a theoretical basis for integrating anatomy, biomechanics, movement science, and systems biology into a unified interpretation of shoulder function.

Table 1 integrates the four organizational domains by summarizing their representative mechanisms, their potential influence on AHD, and their implications for personalized neuromusculoskeletal modelling. Rather than representing discrete biological compartments, these domains should be regarded as complementary analytical perspectives that facilitate the interpretation of multilevel interactions within a single adaptive biological system. This conceptual synthesis provides the theoretical foundation for the discussion that follows, in which the implications of this systems-based framework for shoulder biomechanics, clinical interpretation, and personalized modelling are considered.

Rather than acting independently, these organizational domains interact bidirectionally across functional states, continuously influencing one another and collectively giving rise to the emergent functional state represented by AHD. Figure 2 synthesizes these multilevel interactions and illustrates how AHD may be interpreted as a potential systems-level functional biomarker reflecting the functional organization of the glenohumeral system.

## 3. Discussion

### 3.1. Reinterpreting AHD Beyond Structural Reductionism

The acromiohumeral distance has traditionally been interpreted within a predominantly structural paradigm in which reductions in subacromial space are assumed to reflect pathological alterations associated with rotator cuff dysfunction and shoulder pain [3,4]. This perspective has substantially advanced the understanding of shoulder biomechanics and has strongly influenced both research and clinical practice by promoting the use of AHD as a surrogate indicator of subacromial pathology and mechanical impingement.

However, accumulating evidence indicates that exclusive reliance on structural explanations may be insufficient to fully account for the complexity of shoulder function. Imaging studies and systematic reviews have consistently reported substantial variability in AHD measurements, as well as inconsistent relationships between structural findings, symptoms, and functional performance [9,10]. Similarly, acromial morphology and static anatomical measurements alone demonstrate limited capacity to explain the heterogeneity observed across symptomatic and asymptomatic populations. Recent systematic evidence has further highlighted important heterogeneity in imaging protocols, measurement procedures, and diagnostic thresholds while supporting the interpretation of AHD as a clinically relevant structural and functional biomarker [38].

Rather than challenging the established importance of structural anatomy, the framework proposed in this Perspective places anatomical morphology within a broader systems-based interpretation of shoulder function. From this perspective, structural characteristics define the biomechanical constraints under which shoulder behaviour emerges but do not exclusively determine its instantaneous functional state. Consequently, AHD should be interpreted not simply as a descriptor of anatomical space but as the observable expression of the integrated functional behaviour of the glenohumeral system. Importantly, this reinterpretation should be viewed as complementary rather than oppositional to established biomechanical concepts, extending their explanatory capacity instead of replacing them.

This reinterpretation provides a coherent explanation for several findings that have traditionally been considered difficult to reconcile within reductionist models, including the variability of AHD across different functional tasks, the influence of loading conditions and neuromuscular strategies, and the frequent dissociation between structural imaging findings and clinical presentation. Rather than reflecting contradictory observations, these phenomena may be understood as expected consequences of the adaptive behaviour of a complex biological system.

Accordingly, the principal contribution of the proposed framework is not to replace structural assessment, but to provide a broader interpretive context in which anatomical, connective tissue, neuromuscular, and mechanical determinants are considered as continuously interacting components of shoulder function. This systems-based perspective establishes the conceptual basis for the following discussion on emergence, movement variability, personalized modelling, and future clinical applications.

### 3.2. AHD as an Emergent Functional State Variable

Viewing AHD as an emergent functional state variable fundamentally changes its biological interpretation. Rather than representing an isolated structural measurement, AHD may be understood as a dynamic descriptor of the instantaneous functional state of the glenohumeral system. Within this context, the systems-based framework proposed in this work helps reconcile several longstanding inconsistencies in shoulder research by interpreting AHD as an indicator of system behaviour rather than as an isolated structural metric, thereby providing a potential explanation for the frequently reported discordance between imaging findings, symptoms, and functional performance [9].

From this perspective, AHD reflects the continuous interaction of multiple biological processes operating across structural, connective tissue, neuromuscular, and mechanical levels. Rather than remaining fixed, the functional state represented by AHD continuously adapts to changes in posture, loading conditions, motor control strategies, and task demands. Emerging evidence from motor cortex mapping studies indicates that shoulder muscle recruitment is organized according to functional task demands, reinforcing the concept that shoulder function arises from multilevel neural and biomechanical interactions rather than from fixed anatomical relationships [28]. Accordingly, AHD should be regarded as a state-dependent descriptor rather than as a fixed anatomical attribute.

Consequently, similar AHD values may arise from distinct functional configurations, whereas comparable functional outcomes may be achieved through different structural and neuromuscular pathways. Conversely, similar structural anatomy may generate different AHD values depending on the functional state of the system. Such behaviour is consistent with the concept of biological degeneracy, whereby multiple system configurations are capable of producing equivalent functional outcomes [39]. This perspective helps explain why variability in AHD measurements should not automatically be interpreted as measurement error or pathological instability but may instead reflect the normal adaptive behaviour of a dynamically regulated biological system.

This interpretation aligns with contemporary systems theory, which proposes that biological behaviour emerges from nonlinear interactions among interconnected components rather than from isolated anatomical structures [13,14]. Accordingly, the clinical and biomechanical significance of AHD depends not only on its absolute value but also on the biological context in which it is measured. Measurements obtained under different functional conditions should therefore be interpreted as complementary descriptions of distinct functional states rather than as contradictory observations. This interpretation provides the conceptual foundation for integrating AHD into more dynamic and individualized models of shoulder assessment.

### 3.3. Variability and Context-Dependent Organization

One of the most important consequences of interpreting AHD as an emergent functional state variable concerns the biological meaning of variability. Traditional biomechanical approaches have frequently regarded variability as undesirable noise reflecting poor control or instability. In contrast, contemporary movement science increasingly recognizes variability as a fundamental feature of adaptive biological systems [15,16].

Applied to shoulder biomechanics, this perspective suggests that fluctuations in AHD should not automatically be interpreted as indicators of dysfunction or measurement inconsistency. Instead, changes in AHD may reflect the adaptive reorganization of the glenohumeral system in response to variations in posture, loading conditions, movement demands, and neuromuscular strategies. As illustrated in Figure 2, AHD may therefore be understood as a context-dependent descriptor whose significance emerges from interactions among multiple organizational domains.

The theory of motor synergies further reinforces this interpretation. According to Latash et al. [19], the nervous system stabilizes functional outcomes through coordinated covariation among multiple biomechanical components. Shoulder function may therefore be maintained despite substantial variation in individual structural parameters because functional stability emerges from coordinated system behavior rather than from isolated anatomical characteristics.

This perspective provides a potential explanation for the variability commonly observed in imaging studies and may help clarify why structural measurements alone often fail to predict symptoms, movement quality, or functional capacity. Consequently, variability should not necessarily be interpreted as evidence of reduced measurement reliability but may instead provide valuable information about the adaptive capacity of the shoulder system. From this perspective, repeated AHD measurements obtained under different functional conditions become complementary sources of information that characterize the dynamic behaviour of the glenohumeral system rather than competing estimates of a single anatomical parameter.

### 3.4. Connective Tissue Integration and Biotensegrity

The proposed framework highlights the potential contribution of connective tissue continuity to shoulder organization. Because fasciae, tendons, ligaments, and joint capsules form mechanically interconnected networks, local biomechanical behaviour may be influenced by patterns of force transmission extending beyond individual anatomical structures [20,21]. Consequently, the mechanical environment underlying AHD should be viewed as the product of distributed tissue interactions rather than solely of localized structural relationships.

This perspective extends strictly segmental interpretations of shoulder mechanics by suggesting that alterations in tendon properties, tissue compliance, or myofascial behaviour may influence subacromial organization through mechanisms that cannot be fully explained by static geometric relationships alone. Accordingly, connective tissue continuity provides a plausible mechanical substrate through which local and regional adaptations may contribute to the functional state of the glenohumeral system.

Biotensegrity models offer an additional theoretical perspective by emphasizing the balance between tensile and compressive forces across interconnected structures [22,23]. Although further empirical validation remains necessary, these concepts provide a useful biomechanical framework for understanding how distributed mechanical interactions may contribute to the dynamic regulation of shoulder organization. Within the present conceptual model, biotensegrity is therefore interpreted not as an established explanatory mechanism but as a complementary theoretical construct that may help explain how connective tissue integration participates in the emergence of context-dependent functional states.

### 3.5. Clinical Implications for Shoulder Assessment and Rehabilitation

The reinterpretation of AHD proposed in the present work has important implications for clinical assessment and rehabilitation. If AHD reflects a dynamic functional state rather than a purely structural attribute, identical measurements may have different clinical meanings depending on movement behaviour, neuromuscular control, connective tissue properties, and mechanical demands. Consequently, the clinical significance of AHD cannot be interpreted independently of the functional context in which it is obtained.

This perspective does not diminish the value of imaging but highlights the limitations of relying exclusively on static structural measurements. Although ultrasonographic and radiographic measurements of AHD demonstrate acceptable reliability [1,12], their clinical interpretation remains strongly dependent on testing conditions and may not fully represent functional shoulder behaviour. Accordingly, structural imaging should be regarded as one component of a broader assessment strategy that also considers dynamic movement, neuromuscular performance, and task-specific functional demands.

Consequently, rehabilitation strategies may benefit from greater emphasis on motor control, movement quality, scapular coordination, and task-specific adaptation rather than focusing exclusively on structural correction. Similarly, clinical decision-making may be strengthened by integrating structural imaging with functional assessment, movement analysis, and patient-specific biomechanical characteristics. Rather than replacing structural assessment, the proposed framework supports a more comprehensive interpretation in which anatomical findings are evaluated alongside dynamic functional information to better characterize shoulder dysfunction and guide individualized rehabilitation strategies. This approach is consistent with the broader movement toward personalized musculoskeletal assessment, in which structural, functional, and contextual information are integrated to support clinical decision-making.

### 3.6. Toward Personalized Neuromusculoskeletal Models

**Figure 3:**
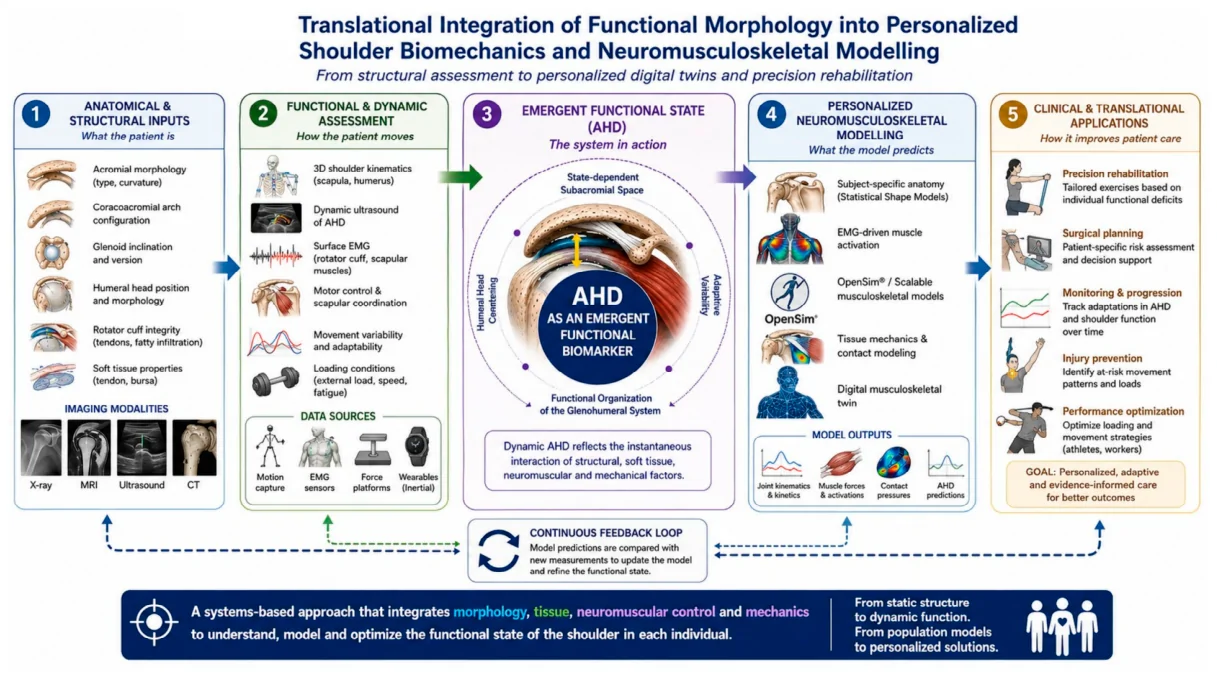
**Translational integration of functional morphology into personalized shoulder biomechanics and neuromusculoskeletal modelling.** Conceptual workflow illustrating how structural anatomy, dynamic functional assessment, and systems-based interpretation of AHD can be integrated into personalized neuromusculoskeletal modelling. The proposed framework supports subject-specific computational models for precision rehabilitation, clinical decision-making, longitudinal monitoring, and future digital-twin applications. Arrows indicate the directional progression and integration of information across the conceptual workflow, while colors distinguish the principal components and stages of the proposed translational framework.

Beyond its immediate conceptual and clinical implications, the proposed framework aligns closely with recent developments in computational biomechanics and personalized neuromusculoskeletal modelling. Advances in subject-specific simulation increasingly allow the integration of anatomy, tissue mechanics, movement behaviour, and motor control within unified biomechanical systems [33,34]. This translational pathway—from structural assessment to personalized modelling and clinical application—is summarized in Figure 3.

Within this context, AHD may represent a potential systems-level functional biomarker capable of linking structural information with individualized functional behaviour. Rather than serving as an isolated imaging parameter, AHD could become an integrative variable connecting structural morphology, movement behaviour, and computational prediction within subject-specific biomechanical models. Such an interpretation is consistent with emerging modelling approaches that seek to capture inter-individual variability in anatomy, tissue properties, movement strategies, and neuromuscular regulation [30,31,32].

The framework proposed here therefore provides a conceptual basis for incorporating multilevel anatomical, biomechanical, and regulatory information into future subject-specific simulation environments. As personalized neuromusculoskeletal modelling continues to evolve, this systems-based interpretation of AHD may contribute to more biologically informed assessment strategies, improved computational prediction, and increasingly individualized approaches to shoulder rehabilitation.

### 3.7. Limitations

Several limitations should be acknowledged. First, the present work is intentionally conceptual and therefore does not provide direct experimental validation of the proposed framework. Although the framework is grounded in existing evidence from anatomy, biomechanics, movement science, and systems theory, experimental and clinical studies will be required to evaluate its assumptions, predictive capacity, and clinical utility.

Second, the complexity of shoulder function implies that additional factors not explicitly incorporated into the present framework—including psychosocial influences, central nervous system adaptations, fatigue-related mechanisms, long-term tissue remodeling, and environmental influences—may also contribute to system behaviour.

Finally, translating systems-based concepts into routine clinical practice remains challenging. The development of practical methods capable of integrating imaging, movement analysis, neuromuscular assessment, and computational modelling will represent an important step toward future clinical implementation.

### 3.8. Future Research Directions

Future research should focus on experimentally evaluating the assumptions of the proposed framework using integrated study designs that combine dynamic imaging, electromyography, motion analysis, computational modelling, and longitudinal clinical assessment. Such approaches may clarify how structural, connective tissue, neuromuscular, and mechanical determinants interact across different functional states, movement tasks, and clinical conditions.

Particular attention should be directed toward identifying state-dependent functional biomarkers capable of capturing changes in shoulder organization across tasks and over time. Longitudinal investigations may further improve understanding of how adaptive and maladaptive organizational patterns emerge during the development and progression of shoulder disorders.

Advances in real-time imaging, wearable sensing technologies, artificial intelligence and emerging digital twin methodologies may facilitate the objective characterization of individualized functional states and provide new opportunities for testing systems-based approaches to shoulder assessment and rehabilitation.

## 4. Conclusions

The present Perspective proposes a conceptual shift in the interpretation of the acromiohumeral distance and shoulder biomechanics. Rather than being viewed solely as a static anatomical measurement, AHD may be more appropriately understood as a systems-level functional biomarker emerging from interactions among structural morphology, connective tissue organization, neuromuscular regulation, movement variability, and mechanical context.

This systems-based functional morphology perspective brings together previously disconnected anatomical and biomechanical evidence into a coherent conceptual framework for understanding shoulder function. By emphasizing these multilevel interactions rather than isolated structural determinants, the framework provides a theoretical basis for understanding why similar anatomical findings may be associated with different functional outcomes and clinical presentations.

Ultimately, interpreting AHD within a systems perspective may help bridge the traditional divide between structural assessment and functional behaviour while providing a conceptual foundation for personalized neuromusculoskeletal modelling, precision rehabilitation, and future subject-specific approaches to shoulder health and disease.

## Figures and Tables

**Table 1:** Multilevel determinants of the acromiohumeral distance and their implications for personalized neuromusculoskeletal modelling.

Organizational Domain	Representative Variables	Potential Influence on AHD	Functional Implications	Potential Implications for Personalized Neuromusculoskeletal Modelling
Structural morphology	Acromial morphology, glenoid inclination, humeral head position, coracoacromial arch configuration	Defines structural constraints and potential subacromial clearance	Influences humeral centering and mechanical space availability	Subject-specific anatomical geometry; joint congruence; structural boundary conditions
Connective tissue properties	Rotator cuff tendon integrity, bursal volume, tissue occupation ratio, tendon stiffness	Modulates dynamic tissue interaction within the subacromial region	Influences load transmission, tissue deformation, and mechanical buffering	Tissue mechanical properties; muscle–tendon parameters; contact mechanics
Neuromuscular regulation	Rotator cuff activation, scapular coordination, proprioception, motor control strategies	Regulates dynamic humeral head stabilization and force distribution	Maintains functional joint centering and movement adaptability	EMG-driven simulations; motor control strategies; muscle coordination models
Mechanical context	Arm position, loading magnitude, movement velocity, fatigue, repetition	Continuously modifies functional subacromial organization	Alters dynamic AHD behavior across functional states	Task-specific simulations; dynamic loading conditions; movement-dependent variability
System-level interactions	Integration between structural, tissue, neuromuscular, and mechanical domains	Generates state-dependent fluctuations in AHD	Determines adaptive or maladaptive functional states	Validation-related biomarker; personalized functional state assessment; digital twin integration

## Data Availability

All extracted and analyzed data are available within the cited studies.
